# Comparison of Flow and Compression Properties of Four Lactose-Based Co-Processed Excipients: Cellactose^®^ 80, CombiLac^®^, MicroceLac^®^ 100, and StarLac^®^

**DOI:** 10.3390/pharmaceutics13091486

**Published:** 2021-09-16

**Authors:** Martin Dominik, Barbora Vraníková, Petra Svačinová, Jan Elbl, Sylvie Pavloková, Barbora Blahová Prudilová, Zdeňka Šklubalová, Aleš Franc

**Affiliations:** 1 Department of Pharmaceutical Technology, Faculty of Pharmacy, Masaryk University, Palackého tr. 1946/1, 612 42 Brno, Czech Republic; martin.dominik@seznam.cz (M.D.); elblj@pharm.muni.cz (J.E.); pavlokovas@pharm.muni.cz (S.P.); 2 Department of Pharmaceutical Technology, Faculty of Pharmacy in Hradec Kralove, Charles University, Akademika Heyrovskeho 1203, 500 05 Hradec Kralove, Czech Republic; vranikovab@faf.cuni.cz (B.V.); svacp3aa@faf.cuni.cz (P.S.); zdenka.sklubalova@faf.cuni.cz (Z.Š.); 3 Department of Physical Chemistry, Faculty of Science, Palacký University Olomouc, Křížkovského 511/8, CZ-771 47 Olomouc, Czech Republic; barbora.blahovaprudilova@upol.cz

**Keywords:** co-processed excipients, spray drying, Cellactose^®^ 80, CombiLac^®^, MicroceLac^®^ 100, StarLac^®^

## Abstract

The utilization of co-processed excipients (CPEs) represents a novel approach to the preparation of orally disintegrating tablets by direct compression. Flow, consolidation, and compression properties of four lactose-based CPEs—Cellactose^®^ 80, CombiLac^®^, MicroceLac^®^ 100, and StarLac^®^—were investigated using different methods, including granulometry, powder rheometry, and tablet compaction under three pressures. Due to the similar composition and the same preparation technique (spray drying), the properties of CPEs and their compacts were generally comparable. The most pronounced differences were observed in flowability, undissolved fraction after 3 min and 24 h, energy of plastic deformation (E_2_), ejection force, consolidation behavior, and compact friability. Cellactose^®^ 80 exhibited the most pronounced consolidation behavior, the lowest values of ejection force, and high friability of compacts. CombiLac^®^ showed excellent flow properties but insufficient friability, except for compacts prepared at the highest compression pressure (182 MPa). MicroceLac^®^ 100 displayed the poorest flow properties, lower ejection forces, and the best mechanical resistance of compacts. StarLac^®^ showed excellent flow properties, the lowest amounts of undissolved fraction, the highest ejection force values, and the worst compact mechanical resistance. The obtained results revealed that higher compression pressures need to be used or further excipients have to be added to all tested materials in order to improve the friability and tensile strength of formed tablets, except for MicroceLac^®^ 100.

## 1. Introduction

The direct compression of active pharmaceutical ingredients (APIs) mixed with different types of excipients is still the most preferred method of tablet preparation. Although this process is time and cost efficient, the insufficient content, mass uniformity, and low mechanical resistance of tablets represent formulation problems that need to be overcome. Single-component excipients often do not provide the required physicochemical properties to allow specific APIs to be adequately formulated by direct compression [1,2]. As a result, the development of new excipients and their combinations has gained increasing attention. Co-processed excipients (CPEs) are solid particulate mixtures of organic or inorganic substances manufactured by different techniques, including spray drying (SD), various types of granulation, melting processes, crystallization, and milling; SD is considered as the most effective way to obtain CPEs of great quality [3].

CPEs tend to have improved physicochemical properties as compared to the simple physical mixtures of components [4]. The main advantages of these solid powder dispersions include uniform particle size and shape distribution, increased density, higher sphericity, and greater porosity; this results in improved flow and compression properties [5]. In addition, they generally have a higher dilution potential, defined as the minimum amount of substance added to the active pharmaceutical ingredient (API), while forming a readily compressible mixture [6]. CPEs are also characterized by their lower sensitivity to lubricants, which is the negative ability of glidants to reduce the compressibility of the resulting mixture [7]. The individual components of CPEs no longer segregate, and their compacts tend to disintegrate faster and sometimes even display improved bioavailability of the formulated drugs [8].

The utilization of CPEs can facilitate tablet manufacturing; therefore, they are manufacturers’ first choice of excipients. For the selection of the most sufficient commercially available CPE, it is extremely important to have a detailed summary of the physicochemical and compression properties of individual CPEs. Although there is basic information available about their physicochemical properties in the form of company literature, these sources often do not provide an extensive overview and comparable values obtained by identical methodology. In addition, available experimental works use mixtures of CPEs with different APIs or lubricants, making the comparison of CPE compression properties inconclusive. For these reasons, the presented work offers an exhaustive comparison of the CPEs themselves; their properties are not burdened by the addition other substances.

CPEs are usually based on three main types of excipients, according to their chemical origin and solubility in water [9]. The first and probably the largest group of CPEs (organic, soluble) is based on sugar alcohols and (poly)saccharides, especially various types of lactose, and rarely contain sorbitol, mannitol, starches, etc. [10]. The second group of CPEs (organic, insoluble) consists mainly of microcrystalline cellulose (MCC) and its various modifications [11]. The last group (inorganic, insoluble) contains substances such as silicates (e.g., magnesium aluminometasilicate) or alkaline earth salts with mineral acids (e.g., calcium and magnesium phosphates and sulphates) [12].

The main aim of this article is to provide a comprehensive characterization of four CPEs, namely Cellactose^®^ 80 (CE), CombiLac^®^ (CO), MicroceLac^®^ 100 (MI), and StarLac^®^ (ST), manufactured by MEGGLE Group GmbH, Wasserburg am Inn, Germany (Table 1) [13]. These CPEs comprise mainly lactose, which makes them representatives of the first and the most important CPE group based on sugars. Lactose is a relatively cheap multifunctional excipient; it is safe to use and available in many forms. As a result, about 60–70% of pharmaceutical dosage forms contain lactose, and thus it represents the major excipient in the pharmaceutical industry [14,15]. In tablet manufacturing, it is usually used as a filler to provide bulk and flow to the formulation and as a binder to give strength to the compact. [14]. However, different static and dynamic flows as well as consolidation behaviors have been detected in various lactose types and preparation methods [16,17]. Lactose is commercially available in two crystalline forms (α, β) and in an amorphous state. While α-lactose occurs in the form of monohydrate and anhydrate, β-lactose exists only as anhydrate [18]. All tested CPEs are prepared by SD technology by drying the aqueous dispersions of lactose and other minor excipients in a stream of hot air while forming a relatively spherical powder in the form of a solid dispersion with good porosity and wettability [19]. The majority of spray-dried lactose spheres contain 80–85% of α-lactose monohydrate crystals, with an admixture of amorphous form and β-lactose. The spray-dried lactose has excellent flow properties, while the amorphous part is used for its better plastic deformation [20]. Moreover, the above mentioned CPEs have relatively uniform size distribution, regular shape, good flow, and, due to the presence of MCC, good compression properties. The manufacturer declares that all these materials are suitable for the production of orodispersible tablets (ODTs), as such dispersion usually disintegrates quickly due to good solubility and improved wettability [14].

ODTs represent a relatively novel oral drug-delivery system, with disintegration within a minute in the mouth due to saliva. The formulation of ODTs improves patient compliance and has additional advantages compared to conventional tablets, such as higher bioavailability. ODTs simplify the oral administration of APIs to pediatric and geriatric patients with swallowing difficulties [21].

The presented work extends the previous studies focused on CPEs such as MCC (Avicel^®^ CE15, Avicel^®^ HFE 102 and Avicel^®^ DG, produced by FMC Health Nutrition, Philadelphia, PA, USA) [22] and inorganic substances (F-Melt^®^ C, F-Melt^®^ M, and F-Melt^®^ F1 from Fuji Chemical Industries Co., Ltd., Toyoma, Japan) [23]. All these materials were comprehensively tested for their flow, viscoelastic, and compression properties. In addition to common pharmacopoeial tests, specific surface areas, electrostatic charge, compact surface using AFM imaging, and other tests were performed to describe overall properties without the addition of API or another excipient (e.g., lubricants), to thoroughly assess properties of pure CPEs. The presented work extends the set of results through a detailed evaluation of lactose-based CPEs and their compacts using sophisticated physical chemistry methods. The used methodology was identical to previous studies, allowing the broader comparison of tested materials and offering tablet manufacturers the opportunity to choose the optimal composition of CPE for their specific purposes.

## 2. Materials and Methods

Cellactose^®^ 80, CombiLac^®^, MicroceLac^®^ 100, and StarLac^®^ were provided by Molkerei MEGGLE Wasserburg GmbH & Co. KG, Wasserburg am Inn, Germany.

### 2.1. Evaluation of CPE Properties

Flow rate, angle of repose, bulk and tapped volumes and densities, Hausner ratio (HR), Carrs’ compressibility index (CI), and pycnometric density were tested in compliance with Ph. Eur. [24]. In addition to pharmacopoeial tests, all CPEs were subjected to the evaluation of particle size distribution (sieve analysis and laser diffraction), specific surface area, angle of slide, moisture content, hygroscopicity, solubility, pH leaching, charge density, differential scanning calorimetry, and surface morphology (scanning electron microscopy) according to the methodology described in detail in the authors’ previous studies [22,23].

The powder rheology of each sample was measured by a FT4 Powder rheometer (Freeman Technology, Tewkesbury, UK). A shear test was applied using the original program “25 mL_Shear_9 kPa”. Samples were loaded into the “25 mL Shear Cell Module” on the FT4 instrument. The weight of the loaded powder was recorded. The initial powder was conditioned using the “Shear Cell Conditioning Module”. After conditioning the powder, the “25 mL Shear Cell Conditioning Module” was replaced with the “24.0 mm Flat-surface Vented Piston”. After pre-consolidation the “Spitting Shim” was pulled forward, and together with the “Slide”, was taken out from the Shear Cell Module. Then the “24.0 mm Flat-surface Vented Piston” was swapped with the “24 mm Shear Cell”. The program was run using the FT4 Powder rheometer. The flow properties of powder were determined as flow function ff.

### 2.2. Compact Preparation, Energy Evaluation of Compaction Process, and Ejection Force 

Cylindrically shaped compacts with a weight of 200.0 ± 1.0 mg and 7 mm in diameter were compressed employing three compression pressures (78, 130 and 182 MPa) using the material testing machine Zwick/Roell T1-FRO (Zwick GmbH, Ennepetal, Germany) equipped with the compaction punches and die (Adamus HT, Machine Factor Group, Szczecin, Poland). During the compression, the energies describing the behavior of powders under compression force were evaluated using a force-displacement record. Immediately after the compression process, the lower punch was removed, and the ejection force was estimated. All methods were performed according to Vodáčková et al. [22] and Svačinová et al. [23].

### 2.3. Evaluation of Compact Properties

Uniformity of mass, friability, hardness, and disintegration time were evaluated in compliance with Ph. Eur. [24] and according to the methodology described in the authors’ previous publications [22,23]. Except for the compendial test, the tablet height and diameter were evaluated and used for the estimation of tensile strength according to Fell and Newton [25].

For a more detailed description of compact properties, the surface roughness and topography (AFM Nanosurf easyScan 2 FlexAFM, Nanosurf, Liestal, Switzerland), pycnometric density (AccuPyc II 1340 Micrometrics, Norcross, GA, USA), wetting time, and water absorption ratio were determined according to the methodology described in Vodáčková et al. [22] and Svačinová et al. [23]. The consolidation behavior of powders and porosity of compacts were calculated according to Equation (1) and Amidon et al. [26], respectively.

(1) $$\mathrm{consolidation}=(V_{b}-V_{t})/V_{b}\times 100$$

where *V_t_* (cm^3^) is the volume of the compact and *V_b_* (cm^3^) is the bulk volume of the same weight of powder (200 mg).

## 3. Results and Discussion

For years, pharmaceutical manufacturers have put much effort into the investigation of directly compressible materials that can facilitate the process of tablet preparation. The CPEs represent a promising group of directly compressible excipients, combining the benefits of commonly used materials. Producers of CPEs usually provide their basic physico-chemical properties, mainly in the form of company literature. However, this information does not allow comparison in the case of different manufacturers, as different evaluation techniques are used to describe behaviors. This study provides a comprehensive evaluation of four commercially available CPEs based on lactose—Cellactose^®^ 80 (CE), CombiLac^®^ (CO), StarLac^®^ (ST), and MicroceLac^®^ 100 (MI). All these materials were manufactured by the SD method; therefore, differences in composition are a crucial factor in distinguishing their behaviors. Compositions of investigated CPEs are summarized in Table 1. CE and MI are identically composed of α-lactose-monohydrate and differ in the form of cellulose presented in their structure (CE—powdered cellulose; MI—MCC). The highest portion of lactose (85%) in combination with maize starch contains ST. CO represents the only tested CPE composed of three substances, namely α-lactose-monohydrate, MCC, and corn starch. The presented work extends previously published studies dealing with the overall characterization of CPEs based on MCC [22] and inorganic compounds [23]. The same evaluation techniques were used, allowing comparison of the properties of CPEs produced by different companies and simplifying the selection process of desired excipients.

### 3.1. Particle Size and Specific Surface Area

**Figure 1:**
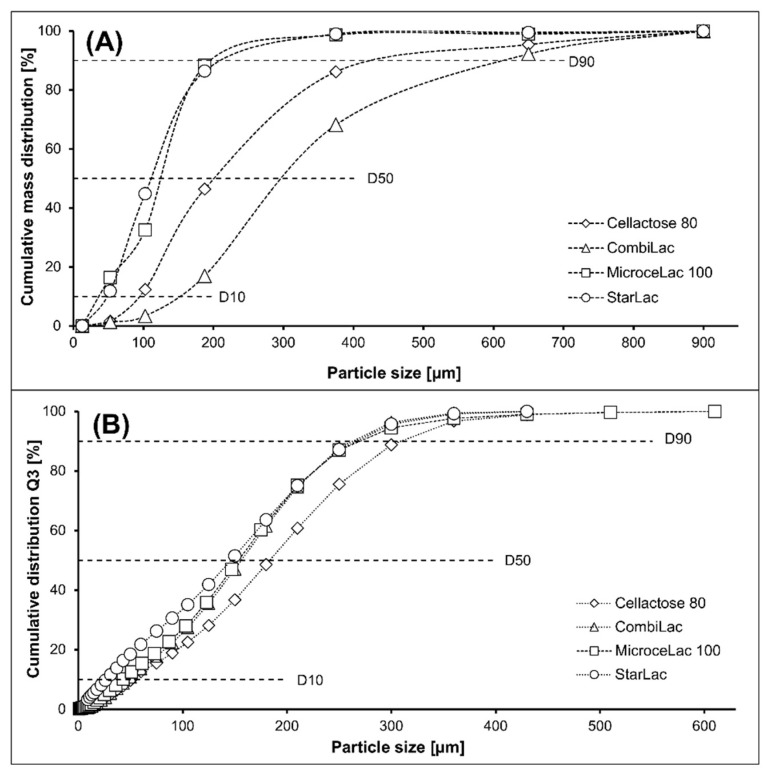
(**A**) Cumulative mass distribution by sieve; (**B**) particle size distribution by diffraction analysis.

The flow properties and miscibility of the tablet mixture are highly dependent on the particle size. The particle size also affects the compression process and quality of the manufactured tablets. The individual components of the powder mixture are more homogenously dispersed if their particle size distribution is comparable. Therefore, knowledge of the particle size distribution of CPEs facilitates selection of the appropriate API particle size [23]. One of the problems resulting from different particle sizes is segregation, which is also affected by the density and shape of the particles. Cellulose and starch tend to have a lower density than inorganic substances, which causes segregation, but small and angular MCC particles make it more difficult for other substances with a higher density to pass through the spaces in the mixture, and thus segregation does not occur. However, major segregation problems may occur for substances with large and spherical particles [2,27]. Using sieve analysis, mean particle size was found to decrease in the following order: CO > CE > MI > ST (Figure 1A). The distribution of size fractions measured by this method was uniform among samples, except MI was divided into two main fractions (fraction 0.025–0.08 mm, forming 24% of the bulk, and fraction 0.125–0.25 mm, forming 59% of the bulk). Comparing the D_50_ parameter, obtained by laser diffraction (Figure 1B), particle size decreased in the following order: CE > MI > CO > ST. A difference in CPE order was caused by different measurement methodologies. When comparing specific surface area (CO, MI > CE > ST) (Table 2), it increased with the amount of cellulose, in particular of MCC, while it decreased with α-lactose-monohydrate in a mixture, confirming the results of Celik and Korpela [28,29]. The relatively high surface area of MCC (about 1.18 m^2^/g) allows its utilization as an adsorbent carrier material in liquisolid systems [30,31]. In contrast, the slow crystallization of lactose produces single crystals with low powder surface area and poor compaction properties [28].

### 3.2. Scanning Electron Microscopy (SEM)

**Figure 2:**
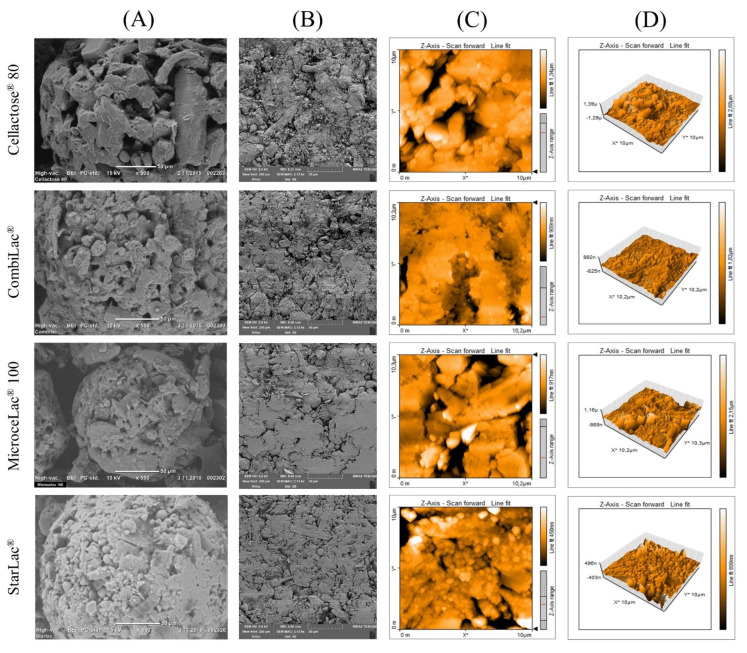
SEM pictures of (**A**) CPE particles (500×, 550×) and (**B**) compacts compressed at 182 MPa. (**C**,**D**) AFM scans of compacts compressed at 182 MPa.

Examined CPEs were manufactured by SD, forming the characteristic spherical and regular shape particles displayed in Figure 2A. In addition, CO and ST are partly formed by unique starch particles [32,33]. Greater porosity is visible for samples containing MCC (CO, MI) and powder cellulose (CE), whose fibers are perspicuous. This observation is consistent with the calculated values of powder porosity discussed below. The general morphology of all studied CPEs is similar due to the dominant content of lactose, which led to comparable powder flow among samples, as discussed above.

### 3.3. Powder Flow

Knowledge of the flow properties of powder is essential for developing a manufacturing process or handling procedure. It is of interest to know how the powder will flow from the hopper during the key situations of tablet manufacturing, as it empties the hopper under gravitation force [34]. In order to evaluate the flow properties of pharmaceutical powders, pharmacopeial and other methods based on powder mobility, such as flow through the orifice, angle of slide, angle of repose, and shear cell, were used (Table 2). The compressibility and changes of powder density were defined using Hausner ratio (HR) and Carr’s index (CI) [35].

The flow through the orifice test can approximate the powder flow rate through a hopper orifice [36]. This method is generally recommended for free-flowable materials rather than cohesive ones. The obtained results showed that the flow properties improved in the following order: MI < CE < CO < ST (Table 2). The best flow properties were measured for ST due to the spherical shape and low porosity of its particles (Figure 2A). According to pharmacopeial testing [24] of the angle of repose, MI and ST exhibited excellent flow, while Cellactose^®^ and CO were characterized as good flowing powders (Table 2). This observation is connected to the spherical shape of all tested CPEs and predicts sufficient mass uniformity of the resulting tablets [37,38]. Angle of slide is a specific parameter typically used to evaluate the flow properties of liquisolid powders and powders with the particle size smaller than 150 μm [30]. Nevertheless, it can also provide information about the powder adhesiveness to the walls of the hopper. Obtained values of angle of slide (Table 2) correlate with the flow through orifice results. Measured values ranged from 28.67 ± 0.58° (CO) to 32.00 ± 1.00° (CE), suggesting good flow properties and a low tendency of all CPEs to remain at the hopper’s walls. The HR and CI are based on the ability of the powder to decrease its apparent density, evaluated by the comparison of bulk and tapped density [22]. Calculated values for both parameters increased in the order CO < ST < MI < CE (Table 2); CO and ST may be classified as “excellent” flowing powders, while CE and MI showed “good” flow according to Ph. Eur. [24]. It is presumed that a stable density of powder through compression results in smaller fluctuations in the mass uniformity of prepared tablets and less pronounced consolidation, as discussed below [39].

A powder material is exposed to consolidation stress during storage, transportation, and the manufacturing process. This exposition changes mechanical interparticulate forces, the voids between particles, and the resulting tapped density of the powder, as clearly demonstrated by the dynamic consolidation of powder lactose by gravity [17]. Measuring the shear properties in the shear test reveal how easily the consolidated powder starts to flow by overcoming its yield point and provides important information about the flowability of the consolidated powder bed. The yield point is affected by the physical properties (size and shape of particles), moisture content, and amount of flow additive [40]. Flow function was used to measure powder flowability, and it increased in the following order: CE < ST < CO < MI (Table 2). All tested materials exceeded the limit for flow function (flow function > 10), establishing all CPEs as free-flowing powders [41].

**Figure 3:**
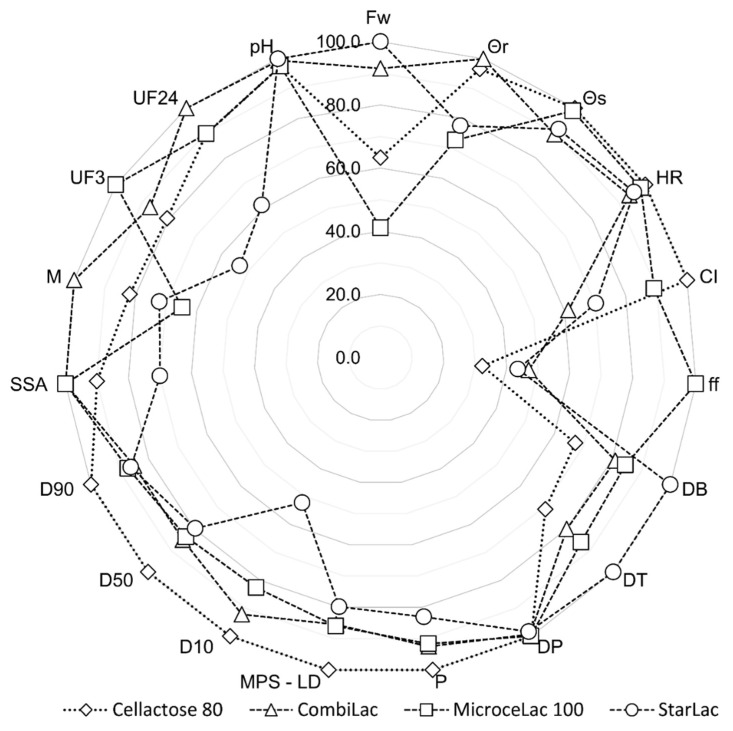
Physical characteristics of CPEs. Fw—flow through the orifice; θr—angle of repose (tg α); CI—compressibility index; DB—bulk density; DT—tapped density; DP—pycnometric density; P—porosity; MPS-LD—mean particle size; D_10_, D_50_, D_90_—the diameters of a sample at the 10th, 50th, and 90th percentiles of the cumulative percent undersize plot; SSA—specific surface area; M—moisture content; UF_3_—undissolved fraction after 3 min; UF_24_—undissolved fraction after 24 h.

All results for powder flow assume that studied CPEs have a good flowability, ensuring uniform die filling with consistent weight uniformity of tablets under production conditions. For a more complex presentation of the flow property results, see Figure 3.

### 3.4. Density and Porosity of Powders

The density of powders is mainly related to dilution potential, flow properties, and the size of tablets. The *bulk* and tapped densities of studied CPEs are in the mid-range of powder classification [42] and correspond to values given by the manufacturer. The pycnometric densities of all CPEs were in the range of 1.543 to 1.568 g/cm^3^, with minimal differences between the materials. The bulk and pycnometric densities were used to calculate the porosity of the powder bed, as porosity is an important factor influencing the flow properties and consolidation behavior of the material (Table 2). The calculated values of the powder bed porosity were in the following order: ST < MI < CO < CE (Figure 3). This observation correlates with the properties of tablets (tablet height and consolidation) discussed below.

### 3.5. Moisture Content, Hygroscopicity, Solubility, and pH Leaching

**Figure 4:**
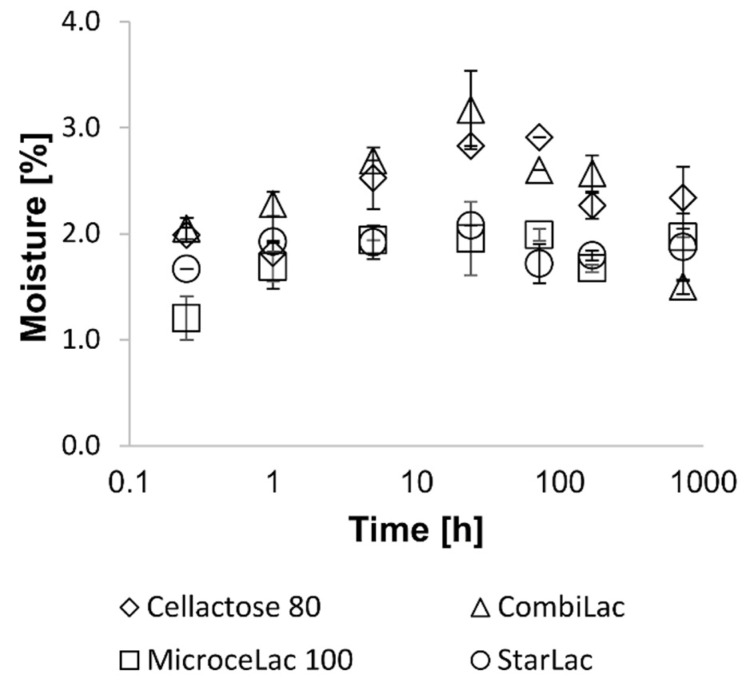
Hygroscopicity of CPEs.

The moisture content of all CPEs was comparable, in the narrow range between 1.14% and 1.76%, slightly increasing in the order MI < ST < Cellactose 80^®^ < CO. ST and CO contain starch that is hygroscopic and rapidly adsorb atmospheric moisture (Table 2). Nevertheless, according to the literature [20,43], corn starch is the least hygroscopic starch, with a relative humidity between 30% and 50%. Hygroscopicity measurements respected the moisture content results, with the highest values for CO containing two hygroscopic substances—starch and MCC (Figure 4).

The solubility test simulates conditions in the oral cavity, with the purpose of determining the amount of insoluble material after dispersion of a tablet in water, which may impact the palatability of the product upon administration. The residue of insoluble material in the mouth is an important parameter in the acceptability of orodispersible formulations [44,45]. The total undissolved fraction after 24 h an increased contra α-lactose monohydrate quantity in the order ST < MI < CE < CO (Table 2). The neutral pH of all the studied CPEs’ dispersions is linked to the content of neutral α-lactose monohydrate, which predisposes these mixtures to be used with a wide range of APIs.

### 3.6. Charge Density

**Figure 5:**
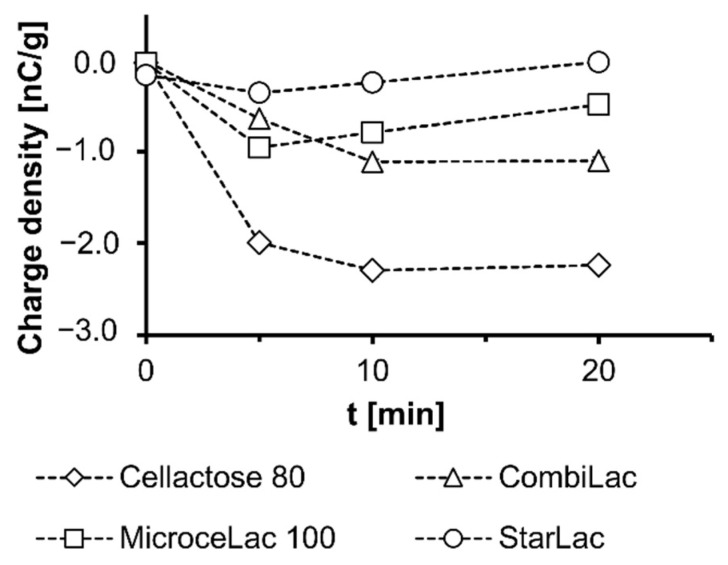
Charge density throughout blending.

Throughout the whole manufacturing and handling process, various interactions occur upon contact or friction among particles of excipients and APIs. These interactions might induce an electrostatic charge in mixtures that affects the formulation, manufacturing process, and packing behavior. Moreover, it can influence the mass and content uniformity of the final product (tablets). For these reasons, the charge density of excipients was examined. All excipients exhibited a negative charge density, as displayed in Figure 5, with the absolute value of charge density halving from CE to CO or MI and being almost zero in the case of ST. The presence of such an electrostatic charge may have an adverse effect on powder blend uniformity. However, as blending of oppositely charged excipients and APIs can lead to a better blend uniformity, all examined excipients might be advantageously used in blends with positively charged APIs. Identical to previously covered CPEs [22,23] of a comparable negative charge, the overall charge density increased strongly in the first minutes and decreased slowly afterwards. Therefore, negatively charged APIs should be preferentially added to blends after pre-blending [22,23].

### 3.7. Differential Scanning Calorimetry (DSC)

**Figure 6:**
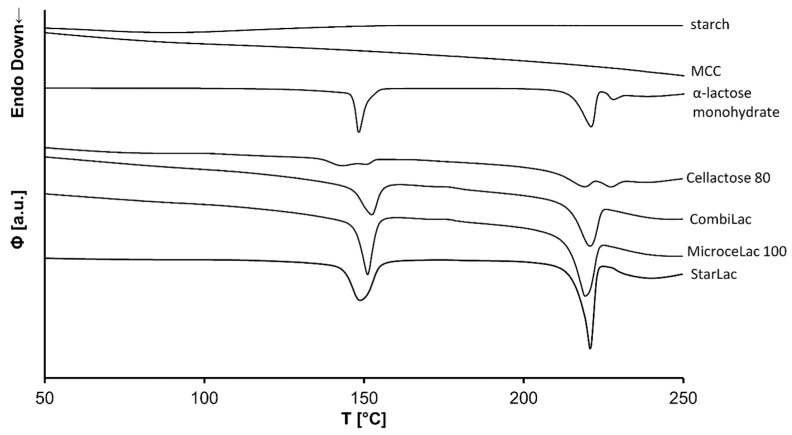
DSC analysis of components and CPEs.

The obtained DSC curves are displayed in Figure 6. The DSC curves of all CPEs show a characteristic endotherm peak in the 130–160 °C region, associated with the loss of crystalline water. This is followed by a peak at 213 °C, corresponding to the melting of α-lactose. Split peaks present at 130–160 °C and 210–230 °C regions in the DSC curve of CE could suggest partial content of β-lactose-anhydrate in this CPE.

### 3.8. Energetic Parameters of Compression Process

Energetic parameters E_1–3_ describe the viscoelastic behavior of powders under the compression pressure. E_1_ expresses the energy consumed for friction and particle rearrangement and is associated with the particle shape and size. This energy should be as small as possible in favor of energy E_2_ [46]. For all measured excipients, the E_1_ energy increases with increasing compression pressure. The highest values were measured for CE, while the lowest were for ST. The obtained values of E_1_ for MI and CO were comparable (Table 3) due to their similar composition. As all excipients are prepared by SD and have almost ideally spherical particles (Figure 2A), the E_1_ energy will be affected mainly by the particle size and surface structure. These parameters also affect the bulk density, which in the case of CE is rather low; therefore, more energy is needed for squeezing out the air trapped between particles and for the rearrangement of particles. In addition, CE has the largest particles, with relatively rough surfaces. The longer fibers of powdered cellulose are not completely incorporated in the structure of particles, and this can lead to higher friction and thus higher values of E_1_ energy [47]. The small differences between CO and MI are related to their particle size distribution, as displayed in Figure 1A,B. MI contains a higher number of smaller particles, and therefore its energy is slightly lower. ST has the smallest particles, and compared to other excipients, also a lower particle surface roughness (Figure 2A). Small particles more easily fill the empty spaces, resulting in reduced friction energy.

The E_2_ energy, accumulated by the tablet during compression, is associated with the die wall friction and plastic deformation of particles [48]. For all excipients, the E_2_ energy increases with increasing compression pressure, while the values decrease at all pressures in the order CE > CO > MI > ST, with only small differences between the first three excipients (Table 3). The differences may be given by the different ratio of lactose, powdered cellulose, or MCC, as well as starch in the composition of CPE. The cellulose particles in the CPE structure undergo plastic deformation, especially at higher compaction pressures [49,50], and form strong interparticle bonds, which are manifested by higher plastic energy [51]. Comparing CO and MI, the slightly higher energy E_2_ in CO can be caused by the presence of starch. In addition to plastic deformation, starch also exhibits more pronounced elastic behavior during compression than cellulose, and thus higher die wall friction, also affecting the ejection force [52,53,54]. In the ST structure, the starch particles are dispersed within the high content of lactose crystals (85%), with low plasticity and weak bonds between particles. Although starch is a plastically deformable material, the plasticity is time-dependent, and it requires a longer time to form strong bonds between particles. A combination of these two factors results in the low plastic energy of ST [55,56].

Energy E_3_ increases with increasing compression pressure; however, the values obtained from the force-displacement record are similar for all used materials. The elasticity of excipients is probably affected mainly by the high content of lactose. Lactose is considered a predominantly fragmenting material, with low elastic deformation being dependent on the compression pressure [57,58,59]. The elastic energy of cellulose and starch is, in the case of the four measured CPEs, probably dispersed by a large number of small lactose particles and thus is not reflected in energy E_3_.

Plasticity shows the deformability of the excipients during compression. The values decrease with decreasing compaction pressure due to the reduced number of compact pores [60]. The values of plasticity for CE, MI, and CO were almost identical, and differences correspond mainly to the energy E_2_ (Table 3).

### 3.9. Ejection Force

The ejection force represents the maximal force needed for the tablet ejection from the die. According to Sun [61], high ejection force is caused by the high residual die wall pressure, resulting in an increase of frictional force. The measured values of the ejection force for the excipients are shown in Table 3. For CE and CO, the ejection force increases with increasing compression pressure; it is higher for CO. In the case of MI, the lowest ejection force was measured at 78 MPa, and the values for higher compression pressures increased but reached comparable values for both pressures (130 and 182 MPa). Moreover, the values of its ejection force are the highest of all tested excipients. For ST, the ejection force first decreased with increasing compression pressure, from 660.83 ± 178.19 N to 550.44 ± 174.85 N, and then increased again to 729.23 ± 87.22 N. Abdel-Hamid and Betz [62] stated that ejection forces are higher for viscoelastic materials compared to brittle ones, which can partly explain the higher values for excipients containing starch and MCC. Generally, the ejection forces are high for all measured co-processed excipients. However, high experimental data variability was observed, resulting in high standard deviations for all excipients and compression pressures. Therefore, the addition of lubricants to all tested materials is needed to decrease the ejection force and make the compaction process smoother.

### 3.10. AFM Imaging

Surface characteristics are essential for liquid absorption (e.g., dissolution medium) or tablet coating adherence, which is an important characteristic, especially for film coating [63]. This measurement can provide quantitative information about irregularities on the compact surface, and in combination with SEM, it is a powerful tool to describe the surface structure of compacts.

The results of surface roughness measurements are shown in Table 4. The RMS parameter for CE, CO, and ST showed almost the same values, while the lowest value was found for MI. However, the differences in compact surface roughness for all measured CPEs are not significant due to the high standard deviations. This can be caused by the composition and structure of compacts. The brittle crystalline lactose fragments during compaction and the small pieces of lactose fill the spaces between larger particles of cellulose or starch. Nevertheless, the cavities are not filled completely, which causes a large number of nonuniform irregularities on the surface (Figure 2C,D). This is detected during surface scanning, and thus it affects the data variability.

Narayan and Hancock [64] stated that the surface roughness can also be related to the mechanical properties of compacts, such as the hardness or friability/brittle fracture tendency. According to their study, brittle materials form smooth brittle compacts with high surface variability, while in the case of ductile powders, the opposite is true. Considering the lactose properties, the surface roughness values, and the standard deviations, the presented results are consistent with this study.

### 3.11. Uniformity of Mass

All prepared compacts fulfilled the requirements given by Ph. Eur. [24] for the uniformity of mass testing, as none deviated from the mean value by more than 7.5% (Table 4). However, the weight variation test was performed only to confirm that all compacts were prepared under the same compaction conditions, as each compact was prepared individually without automatic die filling. Considering the flow properties of all excipients (Table 2) and values of ejection force (Table 3), the deterioration of mass uniformity can be expected when tablets without glidants and lubricants are prepared using an automatic tableting machine.

### 3.12. Pycnometric Density and Porosity of the Compact

As can be seen in Table 4, the pycnometric densities decreased for all used pressures, in the order MI > CE > CO > ST.

The highest density values of MI compacts are connected to the relatively high values of bulk, tapped, and pycnometric density of the powder material (Table 2). No dependency of the compacts’ density on the compression pressure used was observed for this material or for CO. The similarity of pycnometric densities at different pressures may be explained by the decrease in the number of pores available for helium and the simultaneous reduction in the height of compacts with increasing pressures. Moreover, according to Sun [65], the compaction pressure needed to prepare pore-free MCC tablets is 100 MPa. This compression pressure is relatively low for preparing pore-free compacts and is attributed to the high plasticity and good compressibility of MCC and the plasticizing effects of water present in MCC. In contrast, De Boer et al. [66] showed that the pressure necessary for the preparation of pore-free tablets made of lactose is about 450 MPa, due to its fragmentation during the compression process. Considering the composition of MI and CO (Table 1), it can be stated that MCC affects the compression properties of these materials, resulting in the formation of tablets with consistent values of pycnometric density at different compaction pressures. The similarity of these materials is observable also in the mean porosity (Table 2). However, CO showed a more prevalent decrease in porosity values (approx. 12%), with increasing pressures in comparison to MI (approx. 10%). These findings are probably related to the greater reduction in compact height and the consolidation extent of CO.

On the other hand, compacts made of CE that contained powdered cellulose showed a decrease in pycnometric density with increasing compaction pressure. This observation may be explained by the unique structure of this CPE, where the cellulose cores are covered with lactose particles. During the compression, lactose has to undergo fragmentation before the cellulose can be deformed [67]. Subsequently, fragments of lactose fill the interparticle spaces, resulting in tablets with lower porosity and hence lower values of pycnometric density. The increase in compression pressure leads to a greater appearance of lactose fragments and even lower porosity and pycnometric density of compacts. The tablets made of CE showed the highest values of porosity, which decreased with increasing compression pressure, which is in compliance with the results presented by Arida and Al Tabakha [67]. The relatively high porosity of these compacts is related to the low bulk density and higher amount of interparticle spaces between the CE particles and hence a higher amount of air trapped in the compact structure during compression.

The evaluation of ST compacts revealed that an increase in compression pressure causes an increase in pycnometric density. Similar results were observed in previous studies for co-processed materials Avicel^®^ CE15 [22] and F-Melt^®^ F1 [23] and by Sun [63] for tablets containing Avicel^®^ PH102 and are caused by the decreasing height and volume of compacts with increasing compaction pressures. Moreover, compacts made of ST exhibited the lowest values of porosity (Table 4), related to the smaller particle sizes, which better fit together, resulting in decreased interparticle space.

### 3.13. Compact Height and Consolidation Behavior

**Figure 7:**
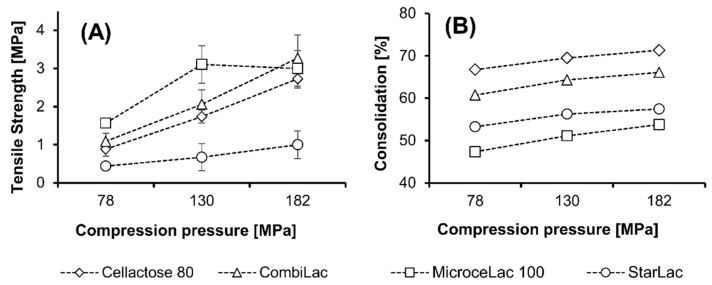
Tensile strength (**A**) and consolidation behavior (**B**).

The compression of all four tested CPEs led to the formation of compacts with similar height. Although CE showed the most pronounced consolidation behavior (up to 71.3%) (Figure 7), related to the lowest value of bulk (0.39 g/mL) and tapped densities (0.42 g/mL), compacts made of this material were the highest compared to other CPEs. The rather low bulk density is related to the higher volume of the powder bed, including the interparticle spaces filled with air. During the compression, the particles need higher energy to be added for their rearrangement, resulting in higher values of energy E_1_ (Table 3). This process is also connected to the formation of higher compacts due to the greater amount of trapped air, resulting in the higher porosity of these compacts, as discussed above. In contrast, the lowest degree of consolidation was observed for MI. The consolidation behavior of this CPE is related to its relatively high values of bulk and tapped density (Table 2), de facto to the low volumes of the powder bed. 

In general, the decrease of compact height with increasing compression pressure (from 78 MPa to 182 MPa) was rather small (9.35–13.20%) in comparison to previously tested CPEs, e.g., Avicel^®^ CE 15 (19.13%) [22] or F-Melt^®^ F1 (17.4%) [23]. This finding may be explained by the composition of different CPEs. All materials tested in this study contain mainly brittle lactose that fragments during compression. These fragments are able to fill the spaces between the particles and fit tighter together, and hence form compacts with a lower height. In contrast, Avicel^®^ CE 15 and F-Melt^®^ F1 contain more elastic materials, e.g., guar gum and MCC in higher concentration, resulting in lower compacts [52,65].

### 3.14. Mechanical Resistance of Tablets

The tensile strength (Figure 7) of prepared compacts decreased in the order MI > CO > CE > ST for all pressures, except 182 MPa, where CO showed the highest compact hardness. As expected, the tensile strength increased with increasing compaction pressure, except for MI, where the moderate worsening of tensile strength occurred with the pressure increment from 130 to 182 MPa. This finding may be related to the fact that CO also contains starch, which requires higher pressures and longer times during compression for the bond’s formation [55]. Similarly, the elastic deformation of starch particles may result in a tensile strength decrease.

The highest increase in tensile strength was observed in compacts containing CO (2.18 MPa), while the lowest was for ST (0.56 MPa). The good compressibility of CO as well as MI, corresponding to the higher tensile strength of their compacts, was also observed in the study presented by Bowles et al. [68], as a result of a combination of plastic (MCC) and brittle (lactose) deforming materials. On the other hand, the rather poor mechanical resistance (low hardness and high friability) of ST compacts is caused by the presence of brittle lactose in the ST composition in a higher percentage (85%) in comparison to other tested materials. ST also comprises maize starch, a plastic material with high elasticity. Therefore, higher pressures and rather lower compression rates are required for the preparation of tablets with sufficient hardness and friability. In addition, the lowest tensile strength of the ST compact corresponds to the lowest E_2_ energy.

The measured values of friability are summarized in Table 4. According to the previous studies testing different CPEs of the Avicel^®^ [22] and F-Melt^®^ [23] type and due to relatively high values of tensile strength, it was expected that all tablets would pass the compendial friability test. Surprisingly, only compacts made of MI using all pressures and compacts containing CO prepared at 182 MPa fulfilled the compendial limit for friability testing (less than 1%). The lower friability of these CPEs is caused by the presence of MCC in their structure, increasing the mechanical resistance of tablets as discussed above. On the other hand, higher concentrations of brittle lactose or more elastic starch led to the formation of tablets with high friability due to more fragile connections between particles. In general, this observation also supports the fact that there is no universal optimal range (e.g., 0.56–1.12 MPa [69]) for tensile strength.

### 3.15. Disintegration Time

All tested materials were designed by the manufacturer as CPEs for direct compression of ODTs [13]. These tablets have to disintegrate rapidly enough to allow swallowing of the formed solution/suspension by patients, and hence maintain their compliance. Therefore, the manufacturers of ODTs often aim at even lower disintegration time, as recommended by the FDA (<30 s) [70], than the one given by Ph. Eur. (<3 min) [24].

The measured disintegration times of compacts are summarized in Table 4. As expected, the compact disintegration slows down with the increasing pressures. All prepared compacts fulfilled the above-mentioned compendial requirement, as their disintegration time was in all cases faster than 2 min. Moreover, compacts made of CE at all pressures, CO at 78 and 130 MPa, and MI at 78 MPa also met the recommendations of the FDA.

The fastest disintegration was observed for CE, which is connected to the highest porosity of its compacts. Additionally, the previously discussed structure of CE [67] can potentiate disintegration, as lactose surrounding the cellulose particles dissolves in the water while forming more pores in the tablet, which leads to faster disintegration.

The obtained results revealed only small differences in disintegration time (Table 4) of ST compacts prepared using different compression pressures. Similar results were noted for the compacts made of CO. Both CPEs contain starch grains, which are deformed under the applied pressures. After moistening by water, the deformed grains regain their original shape, causing the disruption of the compact, whereas the tighter arrangement leads to a greater disintegration, as shown by Lowenthal [71]. Similar results were also described by Hill [72] for corn starch and for co-processed material F-Melt^®^ F1 comprising Fujicalin^®^, MCC, and waxy rice starch [23]. The slightly faster disintegration of CO compacts in comparison to ST compacts is caused by the presence of MCC in the composition of CO. MCC enhances the penetration of water into a compact matrix allowing its faster disintegration [68,73].

The most pronounced effect of pressure on disintegration was noted for MI. The most rapid disintegration (0.27 ± 0.10 min) was observed in tablets compressed by lower pressure (78 MPa), while the tablets prepared by compression pressure of 130 and 182 MPa showed similar disintegration times (around 1.70 min), which is related to the higher values of tensile strength (Figure 7). Though the obtained disintegration times still allow for classification of these compacts, according to Ph. Eur. [24], as rapidly disintegrating, care must be taken if other excipients (especially lubricants) are added to MI. Goto et al. [74] observed that the addition of a lubricant (0.5% of magnesium stearate) can deteriorate the disintegration time to more than 10 min due to its hydrophobic character [75]. A similar observation for the disintegration time (11–14 min) was also reported in tablets containing zolpidem tartrate, MI, Primellose^®^, Aerosil^®^, and magnesium stearate [76]. Therefore, it can be assumed that the addition of hydrophobic lubricants can significantly deteriorate the disintegration time of tablets containing not only MI but also other tested materials.

### 3.16. Wetting Time and Water Absorption Ratio

**Figure 8:**
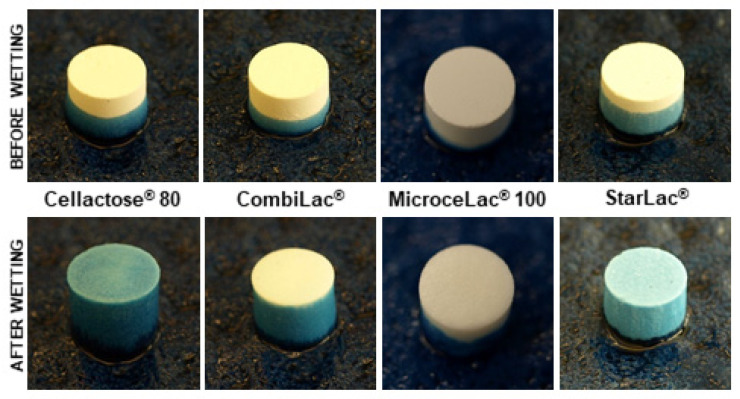
Tablets prepared using a compression pressure of 78 MPa before (*t* = 0 s) and after complete wetting (T2).

The results obtained from the evaluation of wetting times and water absorption ratio are presented in Table 4. The process of wetting in samples prepared using the compression pressure of 78 MPa can be seen in Figure 8. The time needed for the dye solution to reach the upper surface of the compact was lower than 1 min for all compacts prepared using all pressures. The only exception to this observation was compacts made of MI at the highest pressure (182 MPa). The very fast wetting of all compacts is related to the hydrophilic character of all tested CPEs. The other factors that can influence the wetting time of tablets include compaction pressure and the amount and size of tablet pores [77].

Surprisingly, the fastest wetting was observed for ST compacts, even though these compacts showed the lowest porosity. Moreover, the disintegration time of ST compacts was slower in comparison to other tested materials, as discussed above. It was also observed that the wetting time of ST compacts is not affected by the applied compression pressure. All these observations may be explained by the highest concentration of lactose (85%) in the composition of this CPE. Lactose dissolves in contact with water, increasing tablet pore size and hence allowing rapid penetration of water through the whole tablet. This explanation is also supported with the low values of the water absorption ratio. The low water absorption ratio is related to the dissolving process of some components. Similar results were observed for other CPEs containing water-soluble excipients such as mannitol [22,78]. Moreover, the fast wetting time does not allow the starch to start to swell and increase the weight of compacts during wettability testing.

On the other hand, the slowest wetting time was noted for compacts made of MI. These compacts also showed the most pronounced effect of compaction pressure on the time needed for the complete wetting of tablets. The slower wetting in comparison to other tested materials is also related to the slowest disintegration time. This co-processed material consists of lactose with high solubility and microcrystalline cellulose, which allows the penetration of water by wicking [68]. However, the wicking process is significantly affected by the presence of pores in the compact structure and is hence influenced by the compression pressure used. There were pronounced differences in the wetting times of MI and CO, despite their similar composition resulting in mostly similar properties of compacts. The faster wetting time of CO compared to MI compacts is related to lower concentration of MCC and the presence of starch, which enhance the water penetration through the compact.

The compacts made of CE showed, at the lower compression pressure, similar wetting times to ST compacts. However, their wetting is affected by the compression pressure to a greater extent in comparison to ST compacts. The wetting of CE compacts is accompanied by the gentle swelling of powdered cellulose, as can be seen in Figure 8, resulting in a higher value of the water absorption ratio (Table 4).

### 3.17. Comparison with Other CPE Groups

As was mentioned above, manufacturers of directly compressible tablets often face several difficulties (e.g., poorly flowing API, low density and thus high API volume, problematically compressible API, low uniformity of API content in the resulting tablets, and processing of moisture-sensitive API). Therefore, the selection of suitable excipients represents a critical step in the formulation of ODT and directly compressible tablets in general [8,21].

Considering the properties of all tested groups of CPEs, the best flow properties, which are able to compensate for poor flow properties of APIs, have CPEs containing inorganic substances (e.g., magnesium aluminometasilicate), specifically F-Melt^®^ F1. The low density of the API can be equilibrated by the high density of the CPE, based on saccharides and sugar alcohols (e.g., lactose, starch), with ST showing the highest density values. The poor compressibility of the API can be improved by cellulose-based CPEs; Avicel^®^ CE 15 showed the best compaction properties among all tested materials. In addition, the utilization of CPEs based on cellulose leads to the formation of compacts with the best mechanical resistance, including high tensile strength and low friability. Low content uniformity can be the result of many factors, but it often involves a particle size that should be theoretically similar to the particle size of an API. The smallest particles among the tested CPEs were observed for Avicel^®^ DG, while the largest values were noted for CE. For the incorporation of moisture-sensitive ingredients, the F-Melt^®^ CPEs, especially F-Melt^®^ M and F1, are suitable. The disintegration time and wetting characteristics of compacts are of great importance, especially for the formulation of ODTs. The fastest disintegration and wetting times were for lactose-based and F-Melt^®^ CPEs [22,23].

However, it has to be mentioned that this list is not absolute; often it is necessary to assess several issues at once and then to prioritize. In essence, each of the evaluated CPEs can be selected according to the main issue being addressed.

## 4. Conclusions

The flow, viscoelastic, and compression properties of four commercially available co-processed excipients (Cellactose^®^ 80, CombiLac^®^, MicroceLac^®^ 100, and StarLac^®^), prepared by spray drying of lactose with other pharmaceutical excipients, were investigated in this experimental study. As all CPEs are dedicated for direct compression, tablets were prepared under three different compression pressures. To characterize the properties of the co-processed material itself, no glidants, lubricants, or disintegrants were used in order to avoid any additional effect of further excipients. Moreover, the properties of all tested lactose-based CPEs and their compacts were compared with previously published studies describing the properties of co-processed materials based on microcrystalline cellulose (Avicel^®^ CE 15, Avicel^®^ DG, and Avicel^®^ HFE 102) and inorganic substances (F-Melt^®^ C, F-Melt^®^ M, and F-Melt^®^ F1).

In general, the selection of the CPE with the best properties is difficult, as they possess different properties that fit the various needs of pharmaceutical manufacturers. According to the obtained results, it may be stated that the lactose-based CPEs studied in this work are freely flowing materials due to the spherical shape of particles, with a mean particle size in the range of 100–200 nm. In contrast, the ability to deform plastically under pressure (based on E_2_ energy) was much lower in comparison to previously studied CPE materials due to their brittle character, resulting in the lower tensile strength and higher friability of formed compacts. All tested CPEs (except for MicroceLac^®^ 100) require higher compaction pressure or additional excipients to product the tablets fulfilling the pharmacopeial requirements. Moreover, it was noted that all four CPEs exhibited high values of ejection force, suggesting the need for using lubricants to decrease these values and minimize the risk of tablets being damaged during the compression process. As all tested materials are designed as fillers for orally disintegrating tablets, compact disintegration represents the main comparison parameter. The obtained results showed that lactose-based excipients disintegrate rapidly (within 180 s), particularly in comparison to microcrystalline cellulose-based CPEs. The fast disintegration is caused by the greater fraction of highly soluble lactose. The fast dissolving of tablets was confirmed also by the wetting absorption ratio values.

## Figures and Tables

**Table 1:** Composition of CPEs.

**StarLac^®^**	85% alpha-lactose-monohydrate, 15% white maize starch
**Cellactose^®^ 80**	75% alpha-lactose-monohydrate, 25% powdered cellulose
**MicroceLac^®^ 100**	75% alpha-lactose-monohydrate, 25% MCC
**CombiLac^®^**	70% alpha-lactose-monohydrate, 20% MCC, 10% white native corn starch

**Table 2:** Physical characteristics of CPEs.

Measured Value	Cellactose^®^ 80	CombiLac^®^	MicroceLac^®^ 100	StarLac^®^
MPS—LD [µm]	184.05	157.25	158.27	146.8
MPS—SA [µm]	326.11	444.44	203.22	173.43
D10 [µm]	51.39	47.37	42.46	26.80
D50 [µm]	183.41	156.09	153.39	146.21
D90 [µm]	309.20	263.17	269.86	266.7
SSA [m^2^/g]	0.90	1.00	1.00	0.70
Fw [g/s]	11.41 ± 0.05	16.49 ± 0.22	7.40 ± 0.04	18.02 ± 0.12
Θr [^o^]	32.68 ± 0.67	33.85 ± 0.36	24.66 ± 0.56	26.28 ± 0.33
Θs [^o^]	32.00 ± 1.00	28.67 ± 0.58	31.67 ± 0.58	29.33 ± 0.58
HR	1.17 ± 0.00	1.10 ± 0.04	1.15 ± 0.01	1.12 ± 0.01
CI	14.83 ± 0.25	9.08 ± 3.49	13.23 ± 0.78	10.41 ± 0.89
ff	20	47	62	27
DB [g/cm^3^]	0.39 ± 0.00	0.47 ± 0.02	0.49 ± 0.00	0.58 ± 0.00
DT [g/cm^3^]	0.46 ± 0.00	0.52 ± 0.00	0.56 ± 0.00	0.65 ± 0.00
DP [g/cm^3^]	1.568 ± 0.003	1.558 ± 0.001	1.567 ± 0.002	1.543 ± 0.001
P [%]	75.20 ± 0.07	69.56 ± 1.13	68.91 ± 0.26	62.42 ± 0.28
M [%]	1.44 ± 0.04	1.76 ± 0.01	1.14 ± 0.01	1.27 ± 0.19
UF3 [%]	50.21 ± 0.01	53.62 ± 0.01	62.18 ± 0.01	33.08 ± 0.00
UF24 [%]	44.36 ± 0.06	48.60 ± 0.07	44.00 ± 0.16	30.00 ± 0.05
pH	6.69	6.82	6.69	6.86

MPS—LD: mean particle size using laser diffraction; MPS—SA: mean particle size using sieve analysis, D10, D50, D90—the diameters of a sample at the 10th, 50th, and 90th percentiles of the cumulative percent undersize plot; SSA—specific surface area; Fw—flow through the orifice; θr—angle of repose (tg α); θs—angle of slide; HR—Hausner ratio; CI—compressibility index; ff—flow function, DB—bulk density; DT—tapped density; DP—pycnometric density; P—porosity; M—moisture content; UF3—undissolved fraction after 3 min; UF24—undissolved fraction after 24 h; pH—(2% solution).

**Table 3:** Energetic parameters of compression, plasticity, and ejection force.

Measured Value	CP [MPa]	Cellactose^®^ 80	CombiLac^®^	MicroceLac^®^ 100	StarLac^®^
E1 [J]	78	7.57 ± 0.55	6.33 ± 0.33	6.20 ± 0.24	4.54 ± 0.95
	130	13.18 ± 0.95	11.69 ± 0.50	11.42 ± 0.51	8.18 ± 0.39
	182	18.94 ± 1.52	17.68 ± 0.74	16.92 ± 0.83	12.10 ± 0.57
E2 [J]	78	2.66 ± 0.09	2.58 ± 0.03	2.53 ± 0.10	1.80 ± 0.07
	130	4.00 ± 0.17	3.97 ± 0.12	3.78 ± 0.24	2.86 ± 0.13
	182	5.14 ± 0.23	5.00 ± 0.15	4.86 ± 0.28	3.87 ± 0.19
E3 [J]	78	0.24 ± 0.01	0.25 ± 0.02	0.23 ± 0.00	0.26 ± 0.02
	130	0.57 ± 0.01	0.57 ± 0.02	0.56 ± 0.01	0.57 ± 0.02
	182	1.05 ± 0.02	1.05 ± 0.02	1.10 ± 0.04	1.04 ± 0.03
Pl [%]	78	91.68 ± 0.47	91.12 ± 0.54	91.78 ± 0.38	87.22 ± 0.60
	130	87.57 ± 0.59	87.39 ± 0.62	87.01 ± 0.98	83.37 ± 0.07
	182	83.07 ± 0.81	82.64 ± 0.66	81.50 ± 1.25	78.71 ± 1.03
EF [N]	78	321.20 ± 23.39	536.86 ± 67.45	436.60 ± 110.29	660.83 ± 178.19
	130	671.07 ± 30.19	640.52 ± 190.95	777.09 ± 148.31	550.44 ± 174.85
	182	680.31 ± 117.49	770.31 ± 69.05	775.18 ± 111.50	729.23 ± 87.22

CP—compression pressure; EF—ejection force.

**Table 4:** Properties of compacts.

Measured Value	CP [MPa]	Cellactose^®^ 80	CombiLac^®^	MicroceLac^®^ 100	StarLac^®^
UM [mg]	78	198.81 ± 1.50	197.66 ± 7.57	199.78 ± 1.35	199.25 ± 0.93
	130	199.20 ± 0.74	198.08 ± 2.75	200.70 ± 0.34	197.73 ± 1.98
	182	196.96 ± 4.35	198.96 ± 2.22	199.51 ± 0.43	196.48 ± 2.57
	78	-	-	-	-
RMS [nm]	130	-	-	-	-
	182	202.13 ± 122.59	196.08 ± 91.94	130.58 ± 76.30	180.85 ± 119.66
PD [g/cm^3^]	78	1.55088 ± 0.0004	1.54436 ± 0.0001	1.5536 ± 0.0002	1.53477 ± 0.0012
	130	1.54804 ± 0.0004	1.54201 ± 0.0001	1.5501 ± 0.0002	1.53629 ± 0.0009
	182	1.54662 ± 0.0002	1.543001 ± 0.0003	1.5542 ± 0.0002	1.53819 ± 0.0011
Po [%]	78	25.56 ± 2.27	23.45 ± 2.05	23.68 ± 1.21	19.86 ± 1.16
	130	18.66 ± 0.77	15.77 ± 2.34	17.40 ± 1.65	15.06 ± 3.32
	182	15.21 ± 2.86	11.77 ± 1.34	13.23 ± 1.68	13.45 ± 2.01
He [mm]	78	4.46 ± 0.11	4.41 ± 0.09	4.33 ± 0.06	4.28 ± 0.06
	130	4.11 ± 0.03	4.03 ± 0.09	4.02 ± 0.07	3.99 ± 0.15
	182	3.87 ± 0.04	3.83 ± 0.04	3.84 ± 0.07	3.88 ± 0.05
Fr [%]	78	1.83	2.86	0.33	4.79
	130	1.30	3.01	0.15	6.69
	182	3.23	0.83	0.23	5.07
Di [min]	78	0.13 ± 0.00	0.35 ± 0.02	0.27 ± 0.10	0.68 ± 0.07
	130	0.28 ± 0.02	0.37 ± 0.03	1.72 ± 0.70	0.73 ± 0.12
	182	0.48 ± 0.13	0.55 ± 0.03	1.78 ± 0.70	0.72 ± 0.15
T1 [min]	78	0.12 ± 0.00	0.27 ± 0.02	0.70 ± 0.55	0.15 ± 0.02
	130	0.28 ± 0.05	0.25 ± 0.07	0.97 ± 0.33	0.18 ± 0.02
	182	0.97 ± 0.40	0.60 ± 0.35	2.23 ± 1.40	0.15 ± 0.02
T2 [min]	78	0.22 ± 0.03	0.43 ± 0.05	1.25 ± 0.45	0.18 ± 0.00
	130	0.53 ± 0.05	0.65 ± 0.17	2.90 ± 1.32	0.33 ± 0.07
	182	1.65 ± 0.60	1.20 ± 0.55	9.68 ± 5.05	0.28 ± 0.07
WA [%]	78	93.76 ± 3.85	59.59 ± 1.20	63.98 ± 5.75	36.57 ± 4.76
	130	96.65 ± 3.11	66.28 ± 4.65	59.19 ± 5.55	38.83 ± 2.87
	182	101.13 ± 4.75	69.65 ± 2.49	60.90 ± 18.77	37.42 ± 5.28

CP—compression pressure; UM—uniformity of mass; RMS—surface topography and roughness; PD—pycnometric density; Po—porosity of tablets; He—height; Fr—friability; Di—disintegration; T1, T2—wetting time; WA—water absorption ratio.

## Data Availability

The data presented in this study are available on request from the corresponding author. The data are not publicly available due to extensive quantity of values.

## References

[1] Daraghmeh N., Rashid I., Al Omari M.M.H., Leharne S.A., Chowdhry B.Z., Badwan A. (2010). Preparation and characterization of a novel Co-processed excipient of chitin and crystalline mannitol. AAPS PharmSciTech.

[2] Gohel M.C., Jogani P.D. (2005). A review of co-processed directly compressible excipients. J. Pharm. Pharm. Sci..

[3] Miyamoto H. (2008). The Particle Design of Cellulose and the Other Excipients for a Directly Compressible Filler-Binder. KONA Powder Part. J..

[4] Li Z., Lin X., Shen L., Hong Y.L., Feng Y. (2017). Composite particles based on particle engineering for direct compaction. Int. J. Pharm..

[5] Razuc M., Grafia A., Gallo L., Ramírez-Rigo M.V., Romañach R.J. (2019). Near-infrared spectroscopic applications in pharmaceutical particle technology. Drug Dev. Ind. Pharm..

[6] Apeji Y.E., Oyi A.R., Isah A.B., Allagh T.S., Modi S.R., Bansal A.K. (2018). Development and Optimization of a Starch-Based Co-processed Excipient for Direct Compression Using Mixture Design. AAPS PharmSciTech.

[7] Peeters E., Vanhoorne V., Vervaet C., Remon J.P. (2016). Lubricant sensitivity in function of paddle movement in the forced feeder of a high-speed tablet press. Drug Dev. Ind. Pharm..

[8] Franc A., Vetchý D., Vodáčková P., Kubal’ák R., Jendryková L., Gonĕc R. (2018). Co-processed excipients for direct compression of tablets. Ces. A Slov. Farm..

[9] Rojas J., Buckner I., Kumar V. (2012). Co-proccessed excipients with enhanced direct compression functionality for improved tableting performance. Drug Dev. Ind. Pharm..

[10] Saha S., Shahiwala A.F. (2009). Multifunctional coprocessed excipients for improved tabletting performance. Expert Opin. Drug Deliv..

[11] Kaur L., Singh I. (2016). Microwave grafted, composite and coprocessed materials: Drug delivery applications. Ther. Deliv..

[12] Patel H., Patel K., Tiwari S., Pandey S., Shah S., Gohel M. (2016). Quality by Design (QbD) Approach for Development of Co-Processed Excipient Pellets (MOMLETS) By Extrusion-Spheronization Technique. Recent Pat. Drug Deliv. Formul..

[13] MEGGLE. https://www.pharmaceutical-networking.com/meggle-excipients-technology-direct-compression-expertise/.

[14] Hebbink G.A., Dickhoff B.H.J. (2019). Application of lactose in the pharmaceutical industry. Lactose.

[15] Zeman J., Pavloková S., Vetchý D., Pitschmann V. (2021). The effect of different types of lactose monohydrate on the stability of acetylcholinesterase immobilized on carriers designed to detect nerve agents. J. Chem. Technol. Biotechnol..

[16] Hurychová H., Kuentz M., Šklubalová Z. (2018). Fractal Aspects of Static and Dynamic Flow Properties of Pharmaceutical Excipients. J. Pharm. Innov..

[17] Trpělková Ž., Hurychová H., Ondrejček P., Svěrák T., Kuentz M., Šklubalová Z. (2019). Predicting the Angle of Internal Friction from Simple Dynamic Consolidation Using Lactose Grades as Model. J. Pharm. Innov..

[18] Mužíková J., Neprašová M., Faschingbauer H. (2012). Agglomerated Alpha-Lactose Monohydrate and Anhydrous ß-Lactose in Direct Compression of Tablets. Chem. List..

[19] Okáčová L., Vetchý D., Franc A., Rabišková M., Kratochvíl B. (2010). Increasing Bioavailability of Poorly Water-Soluble Drugs by Their Modification. Chem. List..

[20] Sheskey P.J., Cook W.G. (2017). Handbook of Pharmaceutical Excipients.

[21] Dey P., Maiti S. (2010). Orodispersible tablets: A new trend in drug delivery. J. Nat. Sci. Biol. Med..

[22] Vodáčková P., Vraníková B., Svačinová P., Franc A., Elbl J., Muselík J., Kubalák R., Solný T. (2018). Evaluation and Comparison of Three Types of Spray Dried Coprocessed Excipient Avicel^®^ for Direct Compression. BioMed Res. Int..

[23] Svačinová P., Vraníková B., Dominik M., Elbl J., Pavloková S., Kubalák R., Kopecká P., Franc A. (2019). Comprehensive study of co-processed excipients F- Melts^®^: Flow, viscoelastic and compacts properties. Powder Technol..

[24] The European Pharmacopoeia European Pharmacopoeia 9th Edition|EDQM—European Directorate for the Quality of Medicines. https://www.edqm.eu/en/european-pharmacopoeia-ph-eur-10th-edition.

[25] Fell J.T., Newton J.M. (1970). Determination of tablet strength by the diametral-compression test. J. Pharm. Sci..

[26] Amidon G.E., Secreast P.J., Mudie D. (2009). Particle, Powder, and Compact Characterization. Developing Solid Oral Dosage Forms.

[27] Shangraw R.F. (1989). Compressed tablets by direct compression. Pharm. Dos. FORMS Tablets Second Ed. Revis. Expand..

[28] Celik M. (2016). Pharmaceutical Powder Compaction Technology.

[29] Korpela A., Orelma H. (2020). Manufacture of fine cellulose powder from chemically crosslinked kraft pulp sheets using dry milling. Powder Technol..

[30] Vraníková B., Gajdziok J., Vetchý D. (2015). Modern evaluation of liquisolid systems with varying amounts of liquid phase prepared using two different methods. BioMed Res. Int..

[31] Vraníkova B., Gajdziok J. (2015). Evaluation of sorptive properties of various carriers and coating materials for liquisolid systems. Acta Pol. Pharm..

[32] Wu L., Miao X., Shan Z., Huang Y., Li L., Pan X., Yao Q., Li G., Wu C. (2014). Studies on the spray dried lactose as carrier for dry powder inhalation. Asian J. Pharm. Sci..

[33] Walton D.E. (2000). The morphology of spray-dried particles a qualitative view. Dry. Technol..

[34] Faqih A.M.N., Alexander A.W., Muzzio F.J., Tomassone M.S. (2007). A method for predicting hopper flow characteristics of pharmaceutical powders. Chem. Eng. Sci..

[35] Rashid I., Al Omari M.M.H., Badwan A.A. (2013). From native to multifunctional starch-based excipients designed for direct compression formulation. Starch-Stärke.

[36] Ketterhagen W.R., Curtis J.S., Wassgren C.R., Hancock B.C. (2009). Predicting the flow mode from hoppers using the discrete element method. Powder Technol..

[37] Hamzah M. (2018). Beakawi Al-Hashemi, Omar S. Baghabra Al-Amoudi. A review on the angle of repose of granular materials. Powder Technol..

[38] Macho O., Demková K., Gabrišová Ľ., Čierny M., Mužíková J., Galbavá P., Nižnanská Ž., Blaško J., Peciar P., Fekete R. (2019). Analysis of Static Angle of Repose with Respect to Powder Material Properties. Acta Pol. Pharm.

[39] Stanescu A., Ochiuz L., Cojocaru I., Popovici I., Lupuleasa D. (2010). The influence of different polymers on the pharmaco-technological characteristics of propiconazole nitrate bioadhesive oromucosal tablets. Farmacia.

[40] Freeman FT4 Powder Rheometer|Shear Testing|Shear Cell. https://www.freemantech.co.uk/powder-testing/ft4-powder-rheometer-powder-flow-tester/shear-testing.

[41] Freeman R. (2007). Measuring the flow properties of consolidated, conditioned and aerated powders—A comparative study using a powder rheometer and a rotational shear cell. Powder Technol..

[42] Klevan I., Nordström J., Tho I., Alderborn G. (2010). A statistical approach to evaluate the potential use of compression parameters for classification of pharmaceutical powder materials. Eur. J. Pharm. Biopharm..

[43] Wurster D.E., Peck G.E., Kildsig D.O. (1982). A comparison of the moisture adsorption-desorption properties of corn starch, U.S.P., and directly compressible starch. Drug Dev. Ind. Pharm..

[44] Casian T., Bogdan C., Tarta D., Moldovan M., Tomuta I., Iurian S. (2018). Assessment of oral formulation-dependent characteristics of orodispersible tablets using texture profiles and multivariate data analysis. J. Pharm. Biomed. Anal..

[45] Dziemidowicz K., Lopez F.L., Bowles B.J., Edwards A.J., Ernest T.B., Orlu M., Tuleu C. (2018). Co-Processed Excipients for Dispersible Tablets—Part 2: Patient Acceptability. AAPS PharmSciTech.

[46] Voight R., Fahr A. (2006). Pharmazeutische Technologie: Für Studium und Beruf; Mit 109 Tabellen. 10., Überarb. und Erw. Aufl.

[47] Bolhuis G.K., Armstrong N.A. (2006). Excipients for direct compaction—An update. Pharm. Dev. Technol..

[48] Ragnarsson G. (1996). Force-Displacement and Network Measurements. Pharm. Powder Compact. Technol..

[49] Arida A.I., Al-Tabakha M.M. (2008). Compaction Mechanism and Tablet Strength of Cellactose^®^. JJPS.

[50] Govedarica B., Ilić I., Šibanc R., Dreu R., Srčič S. (2012). The use of single particle mechanical properties for predicting the compressibility of pharmaceutical materials. Powder Technol..

[51] Al-Ibraheemi Z.A.M., Anuar M.S., Taip F.S., Amin M.C.I., Tahir S.M., Mahdi A.B. (2013). Deformation and mechanical characteristics of compacted binary mixtures of plastic (microcrystalline cellulose), elastic (sodium starch glycolate), and brittle (lactose monohydrate) pharmaceutical excipients. Part. Sci. Technol..

[52] Ilić I., Govedarica B., Šibanc R., Dreu R., Srčič S. (2013). Deformation properties of pharmaceutical excipients determined using an in-die and out-die method. Int. J. Pharm..

[53] Hauschild K., Picker-Freyer K.M. (2004). Evaluation of a new coprocessed compound based on lactose and maize starch for tablet formulation. AAPS J..

[54] Takeuchi H., Nagira S., Yamamoto H., Kawashima Y. (2004). Die wall pressure measurement for evaluation of compaction property of pharmaceutical materials. Int. J. Pharm..

[55] Anuar M.S., Briscoe B.J. (2009). The elastic relaxation of starch tablets during ejection. Powder Technol..

[56] Adolfsson Å., Olsson H., Nyström C. (1997). Effect of particle size and compaction load on interparticulate bonding structure for some pharmaceutical materials studied by compaction and strength characterisation in butanol. Eur. J. Pharm. Biopharm..

[57] Sebhatu T., Alderborn G. (1999). Relationships between the effective interparticulate contact area and the tensile strength of tablets of amorphous and crystalline lactose of varying particle size. Eur. J. Pharm. Sci..

[58] Zhang Y., Law Y., Chakrabarti S. (2003). Physical properties and compact analysis of commonly used direct compression binders. AAPS PharmSciTech.

[59] Antikainen O., Yliruusi J. (2003). Determining the compression behavior of pharmaceutical powders from the force-distance compression profile. Int. J. Pharm..

[60] Picker-Freyer K.M., Gad S.C. (2008). Tablet production systems. Pharmaceutical Manufacturing handbook Production and Processes.

[61] Sun C.C. (2015). Dependence of ejection force on tableting speed-A compaction simulation study. Powder Technol..

[62] Abdel-Hamid S., Betz G. (2012). A novel tool for the prediction of tablet sticking during high speed compaction. Pharm. Dev. Technol..

[63] Seitavuopio P., Rantanen J., Yliruusi J. (2003). Tablet surface characterisation by various imaging techniques. Int. J. Pharm..

[64] Narayan P., Hancock B.C. (2003). The relationship between the particle properties, mechanical behavior, and surface roughness of some pharmaceutical excipient compacts. Mater. Sci. Eng. A.

[65] Sun C. (2005). True density of microcrystalline cellulose. J. Pharm. Sci..

[66] De Boer A.H., Vromans H., Leur C.F., Bolhuis G.K., Kussendrager K.D., Bosch H. (1986). Studies on tableting properties of lactose—Part III. The consolidation behavior of sieve fractions of crystalline α-lactose monohydrate. Pharm. Weekbl. Sci. Ed..

[67] Arida A.I., Al-Tabakha M.M. (2008). Cellactose^®^ a co-processed excipient: A comparison study. Pharm. Dev. Technol..

[68] Bowles B.J., Dziemidowicz K., Lopez F.L., Orlu M., Tuleu C., Edwards A.J., Ernest T.B. (2018). Co-Processed Excipients for Dispersible Tablets–Part 1: Manufacturability. AAPS PharmSciTech.

[69] Belousov V.A. (1976). Choice of optimal pressure values in tableting medicinal powders. Khimiko-Farmatsevticheskii Zhurnal.

[70] FDA U. (2008). Guidance for Industry Orally Disintegrating Tablets. Fed. Regist..

[71] Lowenthal W. (1972). Mechanism of Action of Starch as a Tablet Disintegrant V: Effect of Starch Grain Deformation. J. Pharm. Sci..

[72] Hill P.M. (1976). Effect of Compression Force and Corn Starch on Tablet Disintegration Time. J. Pharm. Sci..

[73] Yassin S., Goodwin D.J., Anderson A., Sibik J., Wilson D.I., Gladden L.F., Zeitler J.A. (2015). The Disintegration Process in Microcrystalline Cellulose Based Tablets, Part 1: Influence of Temperature, Porosity and Superdisintegrants. J. Pharm. Sci..

[74] Goto K., Sunada H., Danjo K., Yonezawa Y. (1999). Pharmaceutical evaluation of multipurpose excipients for direct compressed tablet manufacture: Comparisons of the capabilities of multipurpose excipients with those in general use. Drug Dev. Ind. Pharm..

[75] Bolhuis G.K., Smallenbroek A.J., Lerk C.F. (1981). Interaction of Tablet Disintegrants and Magnesium Stearate during Mixing I: Effect on Tablet Disintegration. J. Pharm. Sci..

[76] Emshanova S.V., Veselova N.I., Zuev A.P., Sadchikova N.P. (2007). Direct molding technology for the production of zolpidem tablets. Pharm. Chem. J..

[77] Masareddy R., Kokate A., Shah V. (2011). Development of orodispersible tizanidine HCl tablets using spray dried coprocessed exipient bases. Indian J. Pharm. Sci..

[78] Kalia A., Khurana S., Bedi N. (2009). Formulation and evaluation of mouth dissolving tablets of oxcarbazepine. Int. J. Pharm. Pharm. Sci..

